# 肿瘤相关成纤维细胞促进肺癌细胞表达PD-L1

**DOI:** 10.3779/j.issn.1009-3419.2017.05.01

**Published:** 2017-05-20

**Authors:** 海洋 何, 陆玉 齐, 永生 肖, 伊玲 侯

**Affiliations:** 1 300162 天津，武警后勤学院 Logistics University of People's Armed Police Force, Tianjin 300162, China; 2 300162 天津，武警后勤学院附属医院 Hospital Affiliated to Logistics College of Chinese People's Armed Police Forces, Tianjin 300162, China; 3 300162 天津，天津市第四中心医院 TianJin 4th Centre Hospital, Tianjin 300162, China

**Keywords:** 肺肿瘤, 肿瘤相关成纤维细胞, PD-1/PD-L1, 肿瘤免疫逃逸, Lung neoplasms, Tumor-associated fibroblasts, Programmed death factor 1/Programmed death factor ligand 1, Immune escape

## Abstract

**背景与目的:**

肿瘤相关成纤维细胞（tumor-associated fibroblasts, TAF）是肿瘤微环境的重要组成部分，可抑制免疫细胞的功能。在肿瘤免疫中CD8^+^T细胞发挥重要的作用，T细胞膜表面程序性死亡因子1（programmed death factor 1, PD-1），与其配体PD-L1（programmed death factor ligand 1, PD-L1）结合对T细胞的激活起负调节作用。本研究旨在探讨TAF对肺癌细胞PD-L1表达的影响。

**方法:**

我们以肺癌细胞株H1975、H520和TAF细胞进行Transwell非接触式共培养48 h的H1975、H520细胞为实验组，单独培养的H1975、H520细胞为对照组，两组培养条件一致。倒置显微镜计数实验组和对照组H1975、H520细胞数、流式细胞仪分别检测实验组和对照组肺癌细胞PD-L1的蛋白表达率、RT-PCR分别检测实验组和对照组肺癌细胞PD-L1 mRNA的表达。

**结果:**

每100 μm^2^细胞计数，H1975细胞实验组为（46±21）个，对照组为（16±5）个（*P* < 0.05）；H520细胞实验组为（38±10）个，对照组为（12±5）个（*P* < 0.05）。PD-L1蛋白表达率，H1975细胞实验组为（20.93%±3.54%），对照组为（12.58%±2.52%）（*P* < 0.05）；H520细胞实验组（19.26%±3.04%），对照组为（11.60%±2.65%）（*P* < 0.05）。mRNA表达水平，H1975细胞实验组为（16.45±1.25）pg/mL，对照组为（7.78±1.27）pg/mL（*P* < 0.05）；H520细胞实验组为（15.38±2.02）pg/mL，对照组为（7.20±1.58）pg/mL（*P* < 0.05）。

**结论:**

TAF促进肺癌细胞株H1975、H520的生长，增强细胞株PD-L1表达。

## Abstract (en)

**Background andobjective:**

Tumor-associated fibroblasts (TAF) is an important part of TME, which inhibits the function of immune cells. CD8^+^ T cells play a significant role in tumor immunity. T-cell membrane possesses a distinct type of molecule with a negative regulatory function. Upon interaction with its corresponding ligand [programmed death factor ligand 1 (PD-L1)], programmed death factor 1 (PD-1) is activated and thus inhibits the kinase activity of T cells. This study aims to explore the possible effects of TAF on PD-L1 expression in lung cancer cells.

**Methods:**

Lung cancer cell lines H1975 and H520 were co-cultured with (experiment) or without TAF (control) via Transwell assay for through 48 hours under the same culture condition. H1975 and H520 cells were counted using a microscope. The protein and mRNA expression levels of PD-L1 were detected by FCM assay and PCR analysis, respectively.

**Results:**

The numbers of lung cancer cells in 100 μm2 for H1975 and H520 cells are (46±21) and (38±10) in the experiment group, respectively, and (16±5) and (12±5) in the control group, respectively (*P* < 0.05). The expression levels of the PD-L1 protein in H1975 and H520 cells are (20.93%±3.54%) and (19.26%±3.04%) in the experiment group, respectively, and (12.58%±2.52%) and (11.60%±2.65%) in the control group, respectively (*P* < 0.05). The mRNA expression levels in H1975 and H520 cells are (16.45±1.25) and (15.38±2.02) pg/mL in the experiment group, respectively, and (7.78±1.27) and (7.20±1.58) pg/mL (*P* < 0.05) in the control group, respectively (*P* < 0.05).

**Conclusion:**

TAF promotes the growth and increases the expression of PD-L1 in H1975 and H520 cells.

肿瘤细胞可以逃避免疫系统的监视即免疫逃逸，以往的研究主要集中在肿瘤细胞在免疫逃逸中自身的改变，关于肿瘤微环境对免疫逃逸的影响知之甚少。肿瘤相关成纤维细胞（tumor-associated fibroblasts, TAF）是肿瘤微环境的重要组成部分，它不同于正常的纤维细胞，具有特殊的生理、生化特点，可抑制免疫细胞的功能^[1]^。TAF表达跨膜蛋白——成纤维细胞激活蛋白α（fibroblast activation protein α, FAPα），在糖基化形态下具有二肽肽酶及胶原酶活性的同源二聚体。FAPα选择性地表达于90%以上的人上皮性肿瘤，包括乳腺癌、肺癌、结直肠癌及卵巢癌等基质成纤维细胞的包膜或胞浆中，良性及癌前病变的上皮肿瘤中FAPα表达通常为阴性^[2]^（对于非肿瘤组织，FAP表达于胰腺细胞及伤口愈合的基质纤维瘤细胞表面）。

目前已知的TAF细胞的主要功能包括：①细胞表面FAP通过发挥二肽基肽酶和肽链内切酶活性，降解和重建肿瘤与宿主之间的基质，促进肿瘤细胞向胶原底部迁移，从而有利于肿瘤细胞从原发部位脱离，协助癌细胞侵袭和远距离转移^[3]^；②通过产生VEGF、FGF等血管内皮细胞生长因子或招募内皮祖细胞，诱导肿瘤基质中血管内皮细胞网络的形成，促进肿瘤血管生成从而促进肿瘤进展^[4]^；③免疫抑制功能：分泌TGF-β（transforming growth factor-β）、白介素-10（interleukin-10, IL-10）、IL-4、IL-1B以促进Treg（regulatory cells）、肿瘤相关巨噬细胞的生成^[5]^；分泌生长因子、细胞因子、蛋白酶、胞外基质蛋白，干扰T细胞免疫应答；抑制免疫效应细胞。

在肿瘤免疫中CD8^+^ T细胞发挥重要的作用，T细胞的细胞膜上有一类分子对T细胞的激活具有负调节作用，这类分子被称为共抑制分子。主要的共抑制分子包括细胞毒T淋巴细胞相关抗原4（Cytotoxic T-lymphocyte associated antigen 4, CTLA-4）和程序性死亡受体1（programmed death 1, PD-1）。PD-1^[6]^表达于多种细胞表面，在外周遇到相应的配体PD-L1（programmed death factor ligand 1, PD-L1）时被激活，抑制T细胞激酶活性，抑制细胞毒性T淋巴细胞（cytotoxic lymphocyte, CTL）分泌γ-干扰素（interferon-γ, IFN-γ）等细胞因子，同时也可以抑制NK细胞和B细胞的增殖和功能。有研究^[7]^观察到破坏FAP阳性TAF细胞能够激活机体免疫系统，增加IFN-γ和肿瘤坏死因子（tumor necrosis factor, TNF）-α的释放，促进效应T细胞活化，因而本研究旨在探讨TAF对肺癌细胞免疫检点抑制分子PD-L1表达的是否有影响。

## 1 材料及方法

### 1.1 实验材料

人肺癌细胞系H1975、H520购于中科院上海细胞库；人肺癌组织取自已签署知情同意书患者组织样本；胎牛血清购于Gibco，DMEM/F12培养基购于Hyclone，胰蛋白酶购于Sigma；流式抗体PD-L1-PE、Mouse Ig G1 K Isotype Control购于ebioscience；Vimrntin、FAPα、CEA、CA125单克隆抗体购于ABCAM；反转录试剂盒TRIzol购于Life Technologies；SuperScript® Ⅲ反转录酶购于Life Technologies；PCR扩增试剂盒购于TaKara；实验引物来自Quantitech Qiagen；流式细胞仪使用BD LSRII；实时定量PCR使用Bio-Rad iCycler iQ；Transwell小室使用Millicell PISP12R48悬挂式细胞培养皿。

### 1.2 人肺癌肿瘤相关成纤维细胞培养

分离培养并纯化人肺癌相关成纤维细胞：无菌条件下切取肺癌患者手术切除获得的新鲜肺癌组织标本，去除血块及脂肪组织，适量Ⅱ型胶原酶消化成细胞团或单个细胞，终止消化并过200目筛网；转入无菌离心管中，1, 200 rpm离心5 min，弃上清，加入适量含20%小牛血清的DMEM/F12培养液重悬，细胞计数后按1×10^6^个/mL接种于无菌培养瓶，37 ℃、5%CO_2_培养箱中培养。48 h后首次换液，此后每2-3天换液1次。待细胞融合度达到85%，进行细胞传代；自第2次传代起，根据成纤维细胞与肿瘤细胞生长速度及贴壁能力的差异，应用消化法及反复贴壁法纯化细胞，将纯化后的细胞继续培养，每8-10天进行纯化、传代1次。如此反复至第5代。

消化一皿贴壁生长3 d的细胞，1, 000 rpm离心5 min，弃上清，重悬并计数，稀释细胞浓度至1×10^6^个/mL。以100 μL/接种于24孔板，24 h后使用质量分数4%多聚甲醛固定，膜通透后，用山羊血清封闭30 min，加入特异性一抗波形蛋白（Vimentin）、FAPα，癌胚抗原（carcinoembryonic antigen, CEA）、糖类抗原125（carbohydrate antigen 125, CA125），4 ℃孵育过夜，漂洗后加入荧光标记二抗，室温孵育1 h，随后DAPI染核10 min，加入抗荧光淬灭封片剂后，显微镜下观察。试验重复3次。

### 1.3 肺癌细胞复苏与传代

从-80 ℃冰箱中取出H1975、H520冻存细胞，快速将其置于37 ℃水浴中解冻，移至含有6 mL完全培养液的15 mL离心管中，1, 200 rpm离心5 min。弃上清，6 mL完全培养基重悬，接种到T25培养瓶中，37 ℃，5%CO_2_细胞培养箱中培养。第2天，换用6 mL新鲜完全培养基继续培养。待细胞融合度达到85%，进行细胞传代。

### 1.4 共培养体系的建立

取第5代TAF，消化收集制成细胞悬液；分别消化收集传代第2天的H1975、H520细胞制成细胞悬液。设共培养组为实验组，肺癌细胞单独培养组为对照组，培养条件一致。调整TAF细胞密度5×10^5^置于Transwell下室，H1975、H520 3×10^5^置于Transwell上室。24 h细胞贴壁生长，更换上下室培养液。培养24 h倒置显微镜观察细胞生长状态。200倍镜下随机选取5个视野计数细胞数量。

### 1.5 流式细胞仪检测

收集上室实验组H1975、H520细胞与单独培养H1975、H520细胞，4 ℃ 1, 000 rpm离心5 min。弃上清，重悬并计数，稀释细胞浓度至1×10^6^个/mL，每组分别取100 μL×2样本。共培养组设为实验组，单独培养组设为对照组，并设置同型对照组。吸取实验组和对照组100 μL细胞悬液，加入PD-L1-PE抗体2.5 μL，同型对照组加入PE标记鼠IgG1同型抗体，混匀置于4 ℃避光孵育30 min。2 mL PBS洗涤一次，300 μL PBS重悬，样本于BD LSRII检测，各组细胞重复3次。

### 1.6 RNA提取与实时定量RT-PCR

总RNA提取：收集用于流式检测剩余细胞1, 000 rpm离心5 min，弃上清，溶解于TRIzol试剂，具体操作详见说明书。

cDNA合成：1 μg总RNA使用SuperScript® Ⅲ反转录酶反转录，PCR为20 μL体系iQ SYBR Green Supermix，cDNA 1:100稀释。

### 1.7 统计学分析

流式检测数据分析均于FlowJo 9.8软件进行，*Mann Whitney U*检验（GraphPad Prism软件）应用于比较组数据之间的差异。*P* < 0.05为有统计学差异。

## 2 结果

### 2.1 检测分离细胞特定蛋白表达

原代分离肺癌组织样本，纯化、培养5代，免疫荧光染色显微镜观察其具备成纤维细胞相关特性，Vimentin阳性表达（图 1A），同时TAF标志物蛋白FAPα表达阳性（图 1B）；而肺癌相关肿瘤标记物CEA、CA125表达呈阴性（图 1C，图 1D），说明所得细胞为肺癌TAF。

**1:**
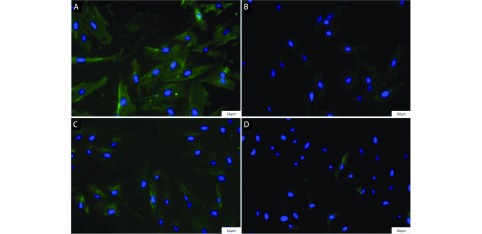
检测分离细胞特定蛋白表达（100×）。TAF标志物Vimentin（A）、FAP*α*（B）表达呈阳性，肺癌相关标志物CEA（C）、CA125（D）表达呈阴性。 Test the specific protein expression of isolated cells (100×). The express of TAF specific proteins Vimentin (A), FAP*α* (B) were positive, while lung cancer related markers CEA (C), CA125 (D) were negative. FAP*α*: fibroblast activation protein *α*; TAF: tumor-associated f ibroblasts; CEA: carcinoembryonic antigen; CA125: carbohydrate antigen 125.

### 2.2 共培养肺癌细胞生长状态

肺癌细胞与TAFs共培养48 h后倒置显微镜观察，实验组肺癌细胞生长密度高，贴壁良好，无明显的脱落、坏死，具有肺癌细胞正常的形态（图 2A，图 2B）。对照组细胞生长密度较实验组低，可见细胞皱缩而不能贴壁而漂浮于培养液中（图 2C，图 2D）。细胞计数结果，H1975实验组每100 μm^2^细胞数为（46±21）个，对照组细胞数为（16±5）个。H520实验组每100 μm^2^细胞数为（38±10）个，对照组细胞数为（12±5）个。

**2:**
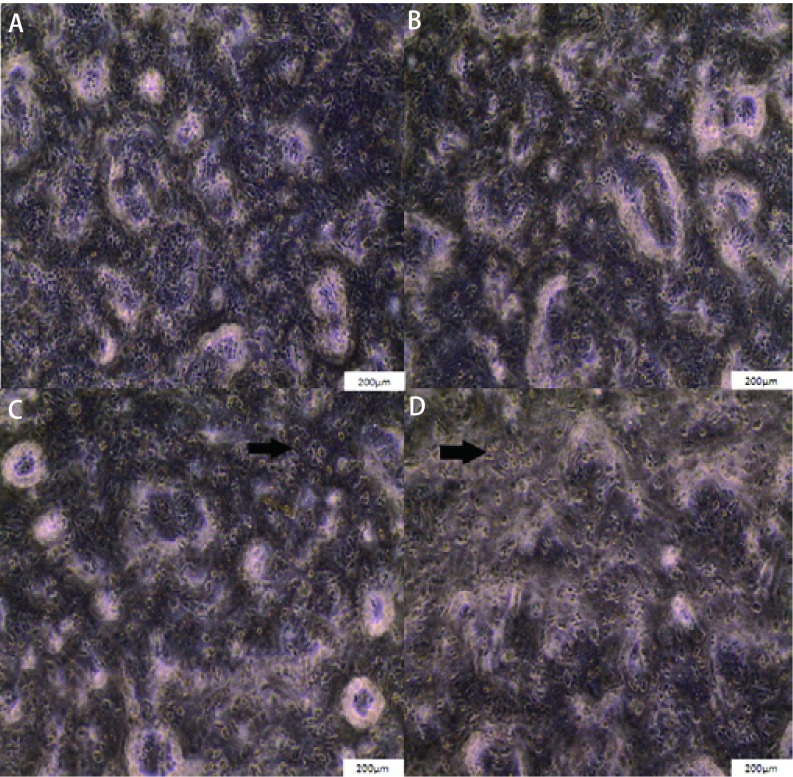
共培养肺癌细胞生长状态。共培养48 h后肺癌细胞（200×）：A：实验组H1975细胞；B：实验组H520细胞；C：对照组H1975细胞；D：对照组H520细胞。箭头位置细胞皱缩不能贴壁而漂浮于培养液中。 Lung cancer cells growth after 48 h. After 48 h co-cultured lung cancer cells (200×): A: Co-cultured H1975 cells; B: Co-cultured H520 cells; C: Control H1975 cells; D: Control H520 cells. Arrows: shrunk cells floating in the culture medium.

### 2.3 肺癌细胞株H1975、H520 PD-L1蛋白表达率

通过流式细胞仪分析共培养及单独培养肺癌细胞悬液，H1975细胞PD-L1蛋白表达率，实验组为（20.93%±3.54%），对照组为（12.58%±2.52%）（*P* < 0.05），H520细胞PD-L1蛋白表达率，实验组（19.26%±3.04%），对照组为（11.60%±2.65%）（*P* < 0.05）。实验组H1975细胞（图 3A）、H520细胞（图 3C）PD-L1表达水平分别较其单独培养对照组（图 3B、图 3D）高。

**3:**
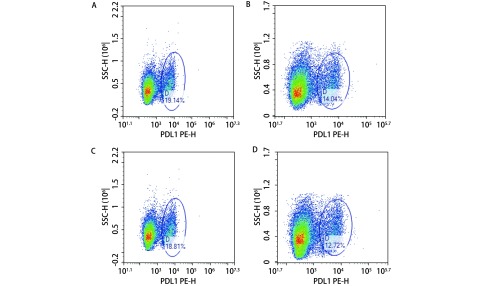
肺癌细胞株H1975、H520 PD -L1蛋白表达率。H1975细胞PD-L1蛋白表达率，共培养组19.14%（A），单独培养组14.04%（B）；H520细胞PD-L1蛋白表达率，共培养组18.81%（C），单独培养组12.27%（D）。 The PD-L1 protein expression in H1975 and H520. The expression of PD-L1 protein in H1975 is 19.14% in the experiment group (A) and 14.04% in the control group (B). The expression of PD-L1 protein in H520 is 18.81% in the experiment group (C) and 12.72% in the control group (D).

### 2.4 RT-PCR分析人肺癌肿瘤相关成纤维细胞对H1975、H520细胞PD-L1 mRNA表达的影响

如表 1所示，H1975细胞PD-L1 mRNA的表达量实验组高于对照组（*P* < 0.05），H520细胞PD-L1 mRNA的表达量实验组高于对照组（*P* < 0.05）。

**1:** H1975, H520细胞PD-L1 m RNA表达 The PD-L1 m RNA expression in H1975, H520

Group	H1975 (pg/mL)	H520 (pg/mL)
Experiment group	16.45±1.25	15.38±2.02
Control group	7.78±1.27^*^	7.20±1.58^*^
^*^: *P* < 0.05.		

## 3 讨论

肿瘤微环境是肿瘤细胞与围绕在肿瘤细胞周围的血管、神经、淋巴管以及上皮细胞、淋巴细胞、巨噬细胞、纤维细胞，以及生长因子、趋化因子、抗体、激酶等多种小分子物质，一起构成的一个庞大网状结构，为肿瘤的发生、发展、侵袭及转移提供条件^[8]^。而免疫系统则在抑制肿瘤的发生、发展过程中起着重要作用，很多免疫缺陷的患者容易发生恶性肿瘤^[9]^，接受免疫抑制治疗的患者肿瘤的发生率也高于正常人^[10]^，其中T细胞介导的免疫应答特别是CD8^+^ CTL发挥重要作用，而T细胞的活化受到PD-1/PD-L1信号通路的抑制。目前已知肿瘤微环境成分之一的TAF可通过多种机制协助癌细胞扩散，基于PD-L1的免疫抑制作用，本实验探索TAF是否能促进肺癌细胞PD-L1的表达。

通过人肺癌组织分离TAF并与2种肺癌细胞H7901（人肺腺癌细胞）、H520（人肺鳞癌细胞）非接触共培养，检测肺癌细胞PD-L1 mRNA及其蛋白的表达。发现共培养组肺癌细胞数多于单独培养组，说明TAF对肺癌细胞的增殖具有促进作用。其次实验组PD-L1蛋白表达率和mRNA相对表达量相对于单独培养组均增加，从而在蛋白和基因水平上证实TAF细胞增加肺癌细胞PD-L1的表达。因采用非接触共培养的模式，我们推断TAF分泌的某些细胞因子可能在这一过程中起关键的作用，具体因子需要在后续实验中进一步探索。

肿瘤细胞表达的PD-L1常被作为预后较差的观测指标^[11]^，针对PD-L1的单抗药物治疗肺癌已获得一定疗效。本实验观察到TAF有助于肺癌细胞PD-L1的表达，因而为治疗肿瘤提供了一条新思路：即将表达FAP的TAF作为靶点，阻断TAF的免疫抑制作用；同时与PD-L1单抗联用，在减少PD-1/PD-L1对CTL细胞的耗竭，增加活性CTL细胞的同时使不表达PD-L1蛋白的肿瘤细胞重新被免疫细胞所识别，利用自身的免疫系统杀死肿瘤细胞。
